# Dr. M. N. Benjamin

**Published:** 1903-01

**Authors:** 


Dr. M. N. Benjamin, of Dunkirk, one of the oldest and best known physicians in that city, died at his home in Central Avenue, November 27, 1902, after a protracted illness, aged 59 years.

Dr. Benjamin was a graduate of Vermont University and served through the civil war as a surgeon in the Union Army, being one of the youngest medical officers in the service. He is survived by a widow and one daughter, Mrs. Frank D. Bliss, of Buffalo.

